# Montmorillonite/Poly(Pyrrole) for Low-Cost Supercapacitor Electrode Hybrid Materials

**DOI:** 10.3390/polym16070919

**Published:** 2024-03-27

**Authors:** Fahim Hamidouche, Zohra Ghebache, Jean-Claude Lepretre, Nacer-Eddine Djelali

**Affiliations:** 1 Laboratory of Polymers Treatment and Forming (LTMFP), M’Hamed Bougara University, Boumerdes 35000, Algeria; f.hamidouche@univ-boumerdes.dz (F.H.); n.djelali@univ-boumerdes.dz (N.-E.D.); 2 Univ. Grenoble Alpes, Univ. Savoie Mont Blanc, CNRS, Grenoble INP, LEPMI, F-38000 Grenoble, France; 3 Faculty of Chemistry, University of Science and Technology Houari Boumediene (USTHB), Algiers 16111, Algeria

**Keywords:** electrical conductivity, controlled capacitance, in situ polymerization montmorillonite, polypyrrole, supercapacitor

## Abstract

Conductive polymers such as polypyrrole have been widely used as pseudo-capacitive electrodes for supercapacitors. This work demonstrates a simple method to improve the performance of conductive polymer electrodes by adding montmorillonite in order to perform capacitive behavior. Conductive composite polymers (CCPs) based on montmorillonite/polypyrrole (MMT/PPy(Cl)) have been synthesized by polymerization using FeCl_3_ as an oxidizing agent. During the preparation of CCP, the effect of MMT/pyrrole mass ratio and the influence of the amount of added H^+^ and temperature of the synthesis medium on the electrochemical performance of the composite have been investigated. The investigation associated with conductivity measurement allowed us to determine the best conditions to reach a high specific capacitance of 465 F gr^−1^ measured by cyclic voltammetry with respect to the CCP synthesized at ambient temperature (220 F gr^−1^) and a 35% increase in capacity compared to its homologue synthesized in neutral conditions at a low temperature. These performances have been advantageously correlated both to the edge acidity of the host material and to the evolution of its conductivity according to the preparation conditions. The galvanostatic charge/discharge tests also confirm the stability of the obtained composite, and a capacitance of 325 F g^−1^ for the best CCP is recorded with a regime of 1 A g^−1^. In addition, the durability of the device shows that the proposed material has a relatively good stability during cycling.

## 1. Introduction

The storage of energy, its use and its recovery in a device depend essentially on the capacity of charge accumulation within the electrode. Carbonaceous compounds were the first materials used in industry for storage of energy in supercapacitors due to their relatively low cost, good stability, large surface area and capacity for the charge accumulation of the electrode/electrolyte interface. However, these materials exhibit some disadvantages such as high internal resistance [1] and large microporous surface, which is relatively inaccessible to the ions of the electrolyte, which reduces their performance and consequently the achievement of low specific capacities of the order 75–175 F g^−1^ for aqueous electrolytes and 40–100 F g^−1^ for organic electrolytes [2].

In this context, researchers have focused on other electrode materials in order to improve the capacitive performance of materials or replace them, in particular, transition metal oxides and electronic conductive polymers [3,4]. These materials exhibit specific capabilities but their disadvantage is their low stability throughout cycling [5]. In this field, the development of binary or ternary CCP electrodes is frequently investigated in order to resolve this problem [6,7].

Conductive polymers exhibit an excellent specific capacitance typically in the range between 100 and 800 F g^−1^ (according to the preparation conditions) [8,9], which is significantly greater than that of the conventional carbon-based electrodes (~100–200 F g^−1^) [10] and comparable to pseudo-capacitive metal oxides [11,12]. However, their structural instability (repeated volumetric swelling and shrinkage during the charge/discharge process) is a major hurdle for their application without omitting their non-negligible progressive degradation throughout cycling [13]. As summarized in Table 1, the literature provides numerous kinds of materials with a wide range of performances.

In this respect, natural clays such as montmorillonite, with a layered structure and large area, are attractive materials as an additive in the electrodes for electric double layer capacitors (EDLCs) [14,15]. In general, the use of clays as electrode material in energy storage systems has been rarely exploited because of their intrinsically low electrical conductivity and low energy storage capacity (70 F g^−1^ in an organic medium) [16,17]. However, this kind of material can play the role of host substrate, allowing the deposition of organic conductive polymer. According to the nature of both host and conductive polymer, experimental conditions can be tuned in order to achieve interesting behavior. The work developed here deals with the association of the capacitive properties of MMT [18,19] with the electrical and pseudo-capacitive properties of polypyrrole. 

**Table 1:** Summary of recent literature on specific capacitance calculated by voltammograms at different scanning speeds (V·s^−1^) of materials for supercapacitors.

Materials/Synthesis Method (C): Chemical (EC): Electrochemical	Electrolyte	Potential Range (V)	Specific Capacitance (F g^−1^)			Number of Electrodes in Cell	Ref.
			0.02 V·s^−1^	0.05 V·s^−1^	0.1 V·s^−1^		
PPy (EC)	0.1 M TBA-ClO_4_	0.0–0.7	50	35	30	Two (S)	[20]
PVF (EC)	0.1 M TBA-ClO_4_	0.0–0.7	25	20	15	Two (S)	[20]
PPy/PVF (EC)	0.1 M TBA-ClO_4_	0.0–0.7	380	350	260	Two (S)	[20]
PMnO_2_ (EC)	1 M Na_2_SO_4_	0.0–1.0	70	60	50	Two (s)	[21]
PGM (EC)	1 M Na_2_SO_4_	0.0–1.0	180	100	50	Two (s)	[21]
PGM/PMnO_2_ (EC)	1 M Na_2_SO_4_	0.0–1.0	480	320	200	Two (s)	[21]
PPy (C)	1 M KOH	0.0–0.9	280	150	100	Three	[22]
PPy/Ni (C)	1 M KOH	0.0–0.9	380	220	105	Three	[22]
CoMnO_2_ (C)	1.0 M KOH	0.0–0.5	350	300	300	Three	[23]
CoMnO_2_/VGCF (C)	1.0 M KOH	0.0–0.5	450	400	320	Three	[23]
PPy/MnO_2_ (C)	1.0 M Na_2_SO_4_	0.3–0.9	40	20	15	Three	[7]
CNT/MnO_2_ (C)	1.0 M Na_2_SO_4_	0.3–0.9	150	125	100	Three	[7]
CNT/PPy/MnO_2_ (C)	1.0 M Na_2_SO_4_	0.3–0.9	285	270	250	Three	[7]
PPy/GO (C)	3.0 M LiCl	0.0–0.6	280	265	250	Three	[24]
NMC (C)	1.0 M H_2_SO_4_	−1.0–0.0	180	170	~155	Three	[14]
MMT	1.0 M H_2_SO_4_		65	26	15	Three	This work
PPy(Cl)	1.0 M H_2_SO_4_		196	126	88	Three	This work
MMT/PPy(Cl)	1.0 M H_2_SO_4_		465	225	130	Three	This work

CNT/PPy: Carbon nanotube/polypyrrole; PVF: Polyvinylferrocene; GO: Graphene oxide; NMC: MMT/carbon (MMT/C) nanocomposites; TBA-ClO_4_: Tetrabutylammonium perchlorate; S: Symmetric.

## 2. Experimental Section

### 2.1. Products, Materials and Analysis Techniques

Pyrrole was bought from Sigma Aldrich (Burlington, MA, USA) with a purity of 98% (CAS number: 109-97-7). The montmorillonite (MMT) originated from Maghnia deposit, Algeria, exhibiting the following XRF massic percentages: SiO_2_ = 50.59%, Al_2_O_3_ = 12.08%, Fe_2_O_3_ = 2.3%, CaO = 0.91%, MgO = 3.08%, fire loss = 31.04%. 

The X-ray diffraction (XRD) analysis of MMT was performed by a Panalytical (Malvern, UK) diffractometer with CuKα radiation (λ = 1.5418 A°, step size: 0.1671°, scanning rate: 0.71 degree.min^−1^). The phases are montmorillonite (M) (Si_3.74_Al_2.03_Fe_0.03_Mg_0.02_O_11_), montmorillonite 15A (M15A) (Ca_0.2_(Al,Mg)_2_Si_4_O_10_(OH)_2_·4H_2_O) and small quantities of quartz (Q) (SiO_2_). The Scherrer equation used in this study is presented as follows (Equation (1)):

(1) $$D=\frac{k\lambda }{\beta cos\theta }$$

where *D* is the average crystalline size, *k* is the Scherrer constant (typically taken as 0.94), *β* is the full width at half maximum (FWHM) of the diffraction peak and *θ* is the Bragg angle [25]. 

The average crystallite size is estimated at 1.699 Ȧ calculated at the most intense peak (5.79°) [25], with an interlamellar space thickness ≈15 Ȧ estimated with the XRD modeling approach [26].

Brunauer–Emmett–Teller (BET) surface area and porosity of MMT were measured by N_2_ adsorption–desorption isotherms at 77 K using micromeritics Gemini Ⅶ. the estimated specific surface area was around 190 m^2^.g^−1^ [27].

Different types of apparatus have been used for the characterization of raw materials and synthesis products, namely: 8400 SHIMADZU (Kyoto, Japan) IRTF spectrometer, ESEM PHILIPS (Amsterdam, Netherlands) XL 30 scanning electron microscope, Panalytical Magix Pro X-ray fluorescence spectrometer to check the chemical composition of MMT and an Autolab potentiometer with Nova 1.7 and 2.1 software.

The experiments were performed in a three-electrode electrochemical cell with an aqueous reference electrode (Ag/AgCl, KCl 0.01 mol.L^−1^), a platinum plate/grid as a counter electrode and a cavity carbon-based electrode as a working electrode. The cyclic voltammetry measurements were performed in the potential range from −0.3 to 0.7 V vs. Ag/AgCl in H_2_SO_4_ 1 mol.L^−1^ aqueous medium, starting with a scanning cathodic potential. 

The voltamperograms presented are those of the second scanning cycle, and the specific capacitance expressed in F g^−1^ is calculated by the following Equation (2):

(2) $$C_{sp}=\frac{1}{mv(v_{2}-v_{1})}\int_{v_{1}}^{v_{2}}I(V)dV$$

where *v*_1_ and *v*_2_ are, respectively, the limits of low and high potential (*V*) and *I* is the instantaneous current (A) on the curves of cyclic voltammetry. *v* is the scan rate (V·s^−1^) and *m* is the mass of the electrode materials (active material) [12].

The galvanostatic charge/discharge technique was used to characterize the performance of the supercapacitor. The geometric charge/discharge capacity *C_sp_* can be calculated from the linear part of the charge/discharge curve by Equation (3):

(3) $$C_{sp}=\frac{I}{m\;(∆V/∆t)}$$

where *I* is the charge/discharge current density, ∆*t* is the charge/discharge time, ∆*V* is the charge/discharge potential window and *m* is the mass of the active material in grams.

The measurement of the electrical conductivity is as follows: the samples are finely ground and compacted under vacuum for 10 min. Then, four pointed probes [28] are placed on a flat surface of the material. A current source imposes a given intensity between the two external points and a potential difference appears between the two internal points. This method makes it possible to overcome parasitic contact resistances between the tips and the material. The application of equal pressure on the four points spaced out on the surface of the sample allowed us to give a precise resistivity measured in samples with different shapes, which can be expressed by the following formula:

(4) $$\varphi =\frac{\varphi ̥}{G₇(W/S)}(Ω\cdot cm)$$

where: φ: electrical resistivity with the correction coefficient [29,30]; S: distance between points (s) = 2.0 mm; W: thickness of the pellet; φₒ: electrical resistivity without correction coefficient:

(5) $$\varphi ₒ=\frac{V}{I}2\pi S$$

where: V: potential difference (V) and I: current intensity (A). The correction coefficient G_7_ (W·S^−1^) is expressed as a function of the thickness of the sample by the following formula:

(6) $$G₇(W/S)=\frac{2S}{W}\ln2$$

According to the Formulas (4)–(6), the resistivity can be expressed as follows:

(7) $$\varphi =\frac{\pi \omega }{\ln2}\frac{V}{I}$$

The conductivity will then be given by the Formula (8):

(8) $$\sigma =\frac{1}{\varphi }\;\;(S/cm)$$

In addition, the conductivity measurement was compared to the optical properties determined by the diffuse reflectance spectrum using a relation between the absorption coefficient (α) and the incident photon energy (hν), which are given by the Tauc [31] relation:

(9) $${\left(\alpha h\nu \right)}^{m}=C(h\nu -E_{g})$$

C is the speed of light in a vacuum. The exponent m depends on the type of transition, m = 2 and ½, respectively, for direct or indirect transitions. The transition is direct in this case.

### 2.2. Synthesis Method

In 50 mL of distilled water containing the well-dispersed MMT, 1.4 mL of pyrrole is added. The amount of MMT is adjusted in order to reach an MMT/pyrrole ratio = 0.1−0.2−0.3−0.4−0.5. After 4 h of stirring, the solution becomes creamy. The polymerization (at room temperature and 0 °C) is initiated by the dropwise injection of 50 mL of a solution of oxidizing FeCl_3_ using the molar ratio FeCl_3_/pyrrole = 2.5 [32]. After 8 h of stirring, the obtained CCPs were filtered, washed with water to remove the iron salt and traces of unreacted pyrrole, then with methanol to extract oligomers, and the yield of the final composite obtained is around 90%.

Finally, the CCP obtained was dried in an oven between 60 to 70 °C for eight hours. For each sample, the purity of the polymer is checked by the absence of the redox signature of the Fe^II^/Fe^III^ system (close to 0.6 V ESH). After each synthesis, we proceeded with characterization of the products, i.e., FTIR, electrical conductivity measurement using the four-probe method and electrochemical measurements, namely cyclic voltammetry (CV) and impedance. The SEM and X-ray diffractogram (XRD) results of the MMT and the CCP are provided in Figures S1 and S2. We attempted to check the impact of the presence of polypyrrole on the interlaminar space of montmorillonite. However, as previously outlined by Borralleras and al. [33], the large polymer coverage leads to a rather difficult determination of the MMT [34].

## 3. Results and Discussion

### 3.1. FTIR Analysis

**Figure 1:**
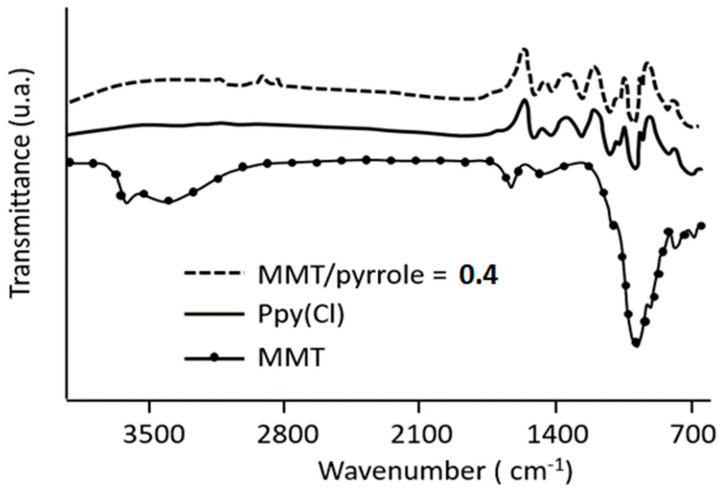
Infrared spectra of MMT, pure PPy(Cl) and MMT/PPy(Cl) synthesized at 25 °C.

The FTIR spectrum of MMT (Figure 1) is consistent with literature results, and the main bands are characteristic of this clay (1000 and 1600 cm^−1^) [35,36]. The FTIR spectrum of pure PPy displays a characteristic peak at 1547 cm^−1^ for 2,5-substituted pyrrole [34] and at 1450 cm^−1^ which can be attributed to the typical vibrations of the pyrrole cycle from polypyrrole. The bands at 1312 and 1028 cm^−1^ may correspond to the vibrations of the =C-H in the plane. The band observed at 930 cm^−1^ for =C-H indicates the presence of polymerized pyrrole, and the bands at 1090 and 838 cm^−1^ can be attributed to the N-C stretching [6]. Concerning the stretching of the composite material, in all cases, the signature of MMT is deeply faded, confirming that MMT is homogenously coated by the organic polymer [37].

### 3.2. Electrochemical Measurements

The aim in this section is to study the influence of the chemical synthesis conditions of CCPs on their electrochemical reactivity, namely the MMT/PPy(Cl) ratio, temperature and HCl concentration in the medium. The electrochemical characteristics of these powders have been investigated by cyclic voltammetry, introducing a small amount of synthesized powder, from about 2 to 4 milligrams, deposited in a carbon-based cavity electrode (diam. 4 mm) which is used as working electrode (all graphs are normalized for 1 milligram of active material). The cyclic voltammogram from −1 to +1.3 V vs. Ag/AgCl for an aqueous 1 molar H_2_SO_4_ solution at 10 mV·s^−1^ scan rate illustrates the stability of the electrolyte in these conditions, since a negligible current value is measured (in the order of ±5 × 10^−4^ A cm^−2^) for an electrode without capacitive material. This allows us to accurately measure the capacitive behavior of the explored composite material. After several measurements, we chose the potential window between −0.3 and 0.7 V which leads to the quasi-rectangular shape of the CV of a supercapacitor.

#### 3.2.1. Influence of Mass Ratio MMT/Pyrrole

**Figure 2:**
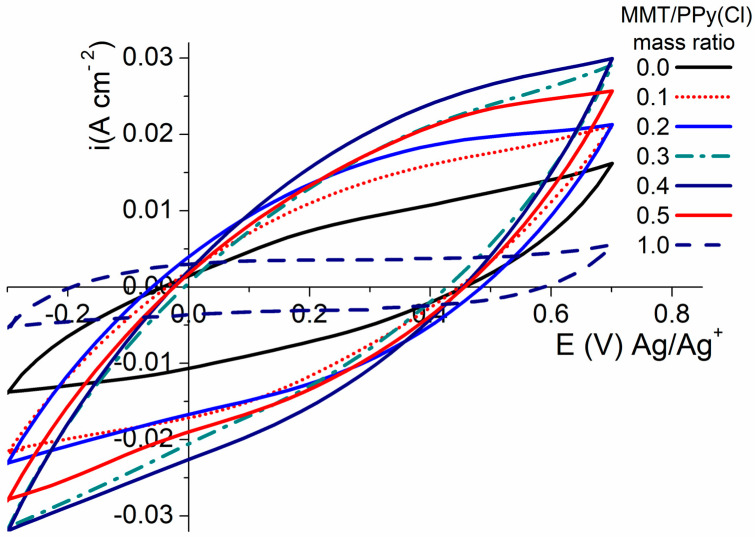
Cyclic voltammetry at 20 mV·s^−1^ of CCP with different mass ratios of MMT/PPy(Cl) synthesized at 25 °C.

Figure 2 shows the electrochemical response of MMT/PPy(Cl) synthesized with different MMT/pyrrole mass ratios. The cyclic voltammograms are recorded with a scan rate of 20 mV·s^−1^ for 10 successive cycles (figures display only the second cycle). The results show a shape similar to that obtained with the carbon-based electrodes [38,39]. In addition, the CVs are not perfectly rectangular and are slightly distorted. One can suggest that this distorted shape originates from electrical resistance appearing at the electrolyte/active material interface. According to the literature, resistance is mainly induced by a heterogeneous distribution of pore sizes. It is also possible to attribute a part of the polarization of the voltamperogram to the low electronic conductivity of the polymer [39].

**Figure 3:**
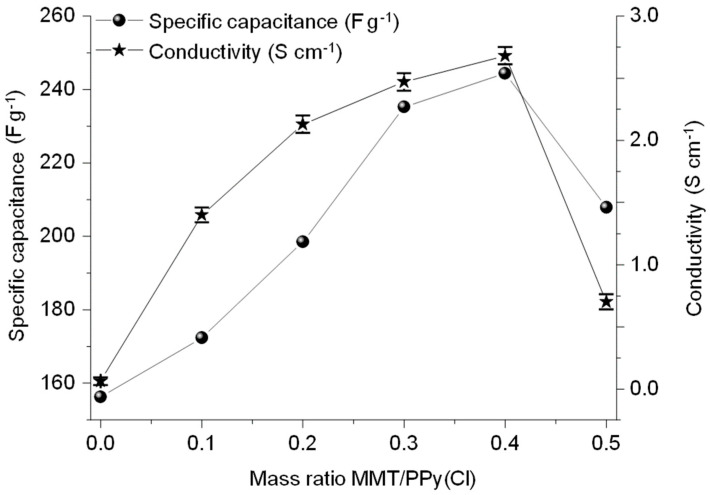
Electrical conductivity (Y1, left axis) and specific capacitance (Y2, right axis) of CCP with different mass ratios of MMT/PPy(Cl) synthesized at 25 °C.

One of the significant results is the specific capacitance (Figure 3), which reaches the value of 245 F g^−1^ with the increase in the MMT/PPy(Cl) mass ratio up to 0.4 (compared to 155 F g^−1^ for the pristine polymer). This improvement with respect to PPy(Cl) can be assigned to the increase in porosity of the overall device and presumably in conductivity, which both could lead to the decrease in the internal resistance. Exploring a higher mass ratio, a decrease in specific capacitance is observed. This result shows that the specific capacitance is in good agreement with the electrical conductivity of the material since the conductivity and the capacitance evolutions vs. MMT/PPy(Cl) ratio share a similar shape.

The electrical conductivity increases with the increase in the amount of MMT up to the ratio 0.4 to reach a value of electrical conductivity of 2.68 S cm^−1^. For the highest MMT/pyrrole ratio, a decrease in conductivity is observed. This can be attributed to the phenomenon of saturation of PPy(Cl) with MMT (the presence of a large amount of MMT in the matrix significantly reduces the electrical conductivity). The same MMT/pyrrole ratio was observed by Peikertovaa et al. regarding the conductivity of the resulting composite [40].

#### 3.2.2. Influence of the Temperature on the Electrochemical Performance 

The impact of the synthesis temperature was examined for a 0.4 MMT/pyrrole ratio. Whereas at 25 °C the capacity is 245 F g^−1^, lowering the medium temperature to 0 °C during the composite preparation leads to a significant increase in the capacity, reaching around 310 F g^−1^ [41]. This result is in good agreement with the literature in which it was proposed that, at a relatively low temperature, the lower kinetic polymerization reduces the contribution of defects within the polymeric structure. This result is in good correlation with the increase in the electrical conductivity since, by reducing the temperature, σ increases by 16% with respect to the conductivity measured for a composite prepared at 25 °C (3.08 vs. 2.68 S cm^−1^) [14]. It also means that mainly α–α bonds occurred during low-temperature polymerization with respect to a few α–β bonds of pyrrole rings whose contribution leads to lower conductivity.

**Figure 4:**
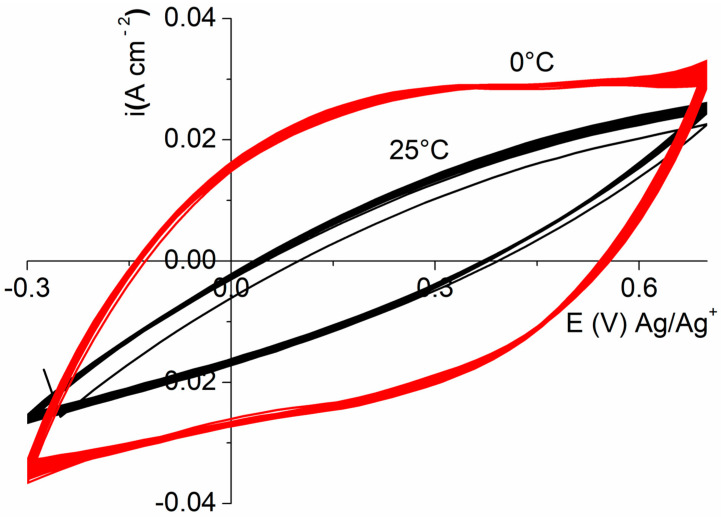
Cyclic voltammograms (100 cycles) of MMT/PPy(Cl) composite (mass ratio = 0.4) synthesized at 0 and 25 °C with scan rate of 20 mV·s^−1^.

Figure 4 displays that this high capacity associated with a great stability during cycling allows reaching a stable value of 297 F gr^−1^ for the material synthesized at a low temperature after 100 cycles at a 20 mV·S^−1^ scan rate (95% retention) (Figure S5) with a stable capacity value after 50 cycles. A similar retention is also observed for the CCP obtained at 25 °C with a value of 233 F gr^−1^ but an unstable capacity value until the end of cycling. This great stability at 0 °C demonstrates the presence of a better structure of the CPP material fixed at a low polymerization temperature with structural conservation throughout the cycling.

#### 3.2.3. Effect of pH on the Performance of the Conductive Composite Polymers

**Figure 5:**
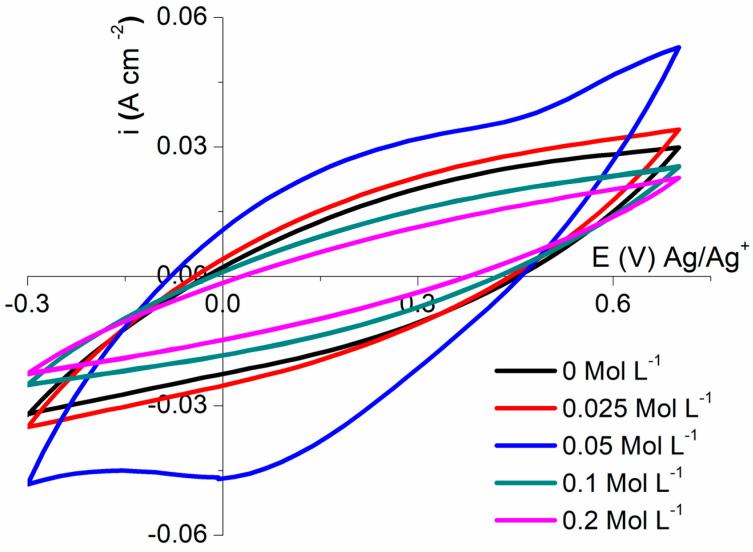
Cyclic voltammetry at 20 mV·s^−1^ of MMT/PPy(Cl) composite (mass ratio = 0.4) synthesized at 0 °C with various concentrations of HCl.

Knowing that 0 °C improved the electrochemical performances of the MMT/(PPy(Cl) conductive composite polymers, we investigated the impact of the amount of added HCl in the synthesis medium on the electrochemical characteristic of the supercapacitor material in order to tune the ionic character of the edge surface of MMT. Figure 5 displays the influence of HCl concentration on the electrochemical performances of the CCP. One can observe that the capacity significantly increases up to 0.05 mol L^−1^ HCl to reach a capacity value of 465 F g^−1^ (vs. 297 F g^−1^ in the absence of acid). On the other hand, increasing the HCl concentration up to 0.2 mol.L^−1^ leads to a decrease in the performance of the material (i.e., 220 F gr^−1^ at 0.2 M).

The decrease in the specific capacitance is presumably due to less favored adsorption of pyrrole in the presence of acid. However, for MMT, pKa values are difficult to accurately determine, particularly due to the fact that the edge site Si-OH and Al(OH)_2_ seem to exhibit similar pH behavior. However, according to the literature, the Si-OH function is deprotonated in neutral conditions (anionic surface), whereas the protonation of these sites (cationic surface) can be observed in strong acidic conditions [42,43]. As illustrated by Brady et al. [44], the protonation of silanol groups at edges is not probable above pH ∼3.5, since silica surfaces are anionic down to pH ~3. However, the contribution of silanol groups to the base consumption and negative charge formation in a deprotonation reaction $\left(Si-OH⇋Si-O^{-}+H^{+}\right)$, especially above pH ~8, may be dominant. Knowing that, it can be suggested that in the presence of HCl, some sites are progressively protonated according to the amount of added HCl. The progressive cationic character of the surface, increasing its hydrophilic character, leads to a less favored adsorption of the lipophilic pyrrole prior to polymerization [45]. In addition, this lower performance can be due, as well, to the formation of (2,5-bis (2-pyrrolyl) pyrrolidine as a side product in an acidic medium which leads to saturated bridges between polypyrrole moieties, thus reducing the overall conductivity of the polymer [46].

Comparing the conductivity of the composite, a higher value is measured at 0.05 molL^−1^ HCl (5.04 S cm^−2^), whereas, when increasing the amount of acid, the conductivity values drop down (2.38, 2.06 S cm^−1^ for 0.1, 0.2 mol L^−1^ HCl, respectively). This result is in good agreement with previous results which show that the side formation of insulator 2,5-bis (2-pyrrolyl) pyrrolidine [46,47] is enhanced when polymerization is performed at a pH lower than 1 (Figure S3). 

The intersection of the linear (αhν)^2^ system plot with the hν axis (Figures S4 and S5) gives a direct optical transition (optical) energy gap. The gap energy is 1.0 eV for MMT/PPy(Cl) which is lower than that of the pure PPy(Cl) (i.e., 1.35 eV), which in good accordance with conductivity measurements, i.e., the smaller the gap, the higher the conductivity of the material.

**Figure 6:**
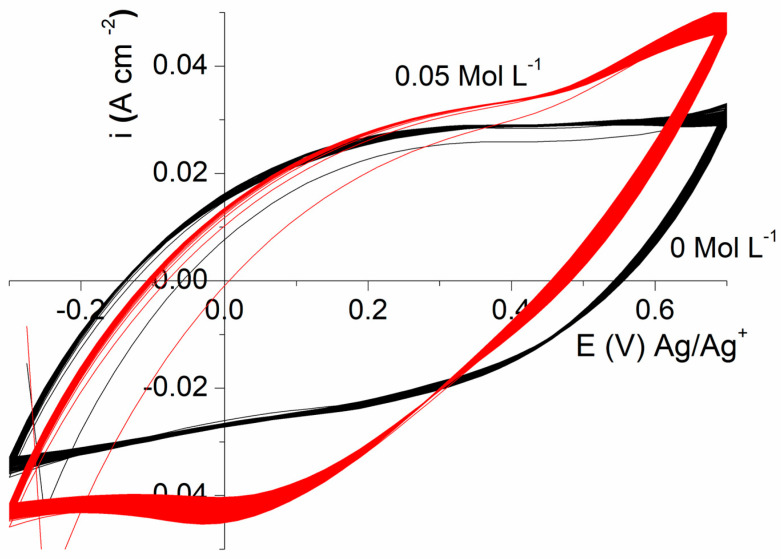
Cyclic voltammetry (100 cycles) at 20 mV·s^−1^ of MMT/PPy(Cl) composite (mass ratio = 0.4) synthesized at 0 °C with a concentration 0 and 0.0 5 M HCl.

Concerning the preservation of the performance, numerous cycles have been performed according to the amount of added HCl (Figure 6). 

**Figure 7:**
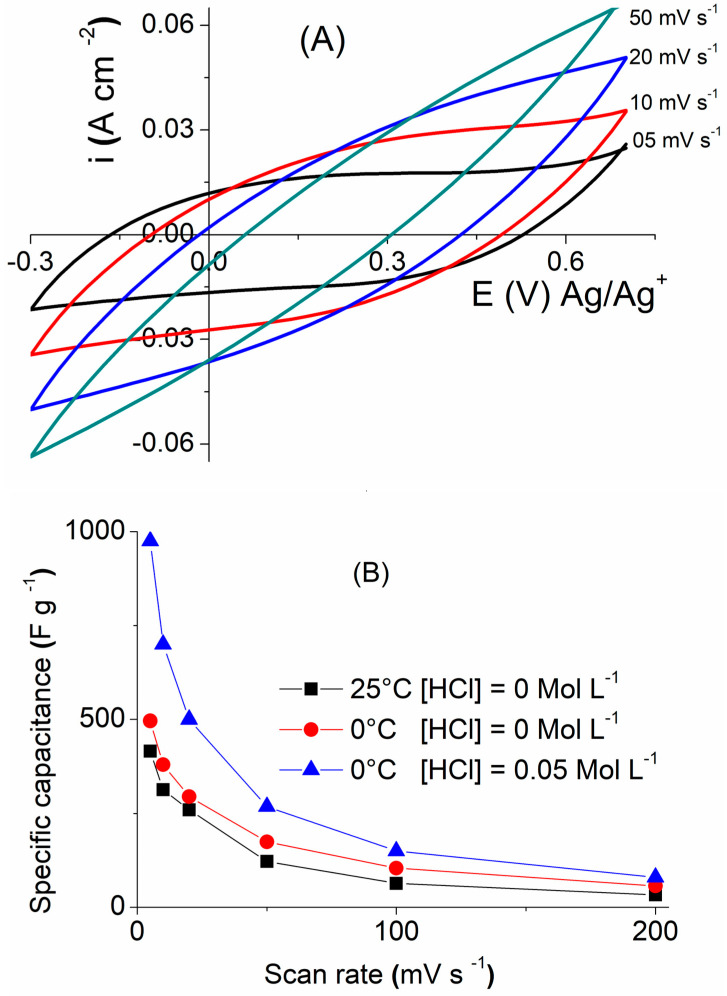
(**A**) Scanning cyclic voltammetry of 100 to 5 mV·s^−1^ of MMT/PPy(Cl) composite (mass ratio = 0.4) synthesized at 0 °C and 0.05 M HCl. (**B**) Specific capacitance of MMT/PPy(Cl) composite (mass ratio = 0.4) synthesized at 0 °C and 25 °C and with or without 0.05 M HCl.

Although in the presence of HCl 0.05 mol.L^−1^, the capacity of the composite slightly decreases, with respect to the one measured in the absence of HCl, more than 88% of the initial capacity is preserved after 75 cycles while, for the 100 subsequent cycles (Figure S5), the capacity remains almost unchanged with a final stable value of 409 F g^−1^ (Figure 7). It has to be outlined that these values are significantly greater than the ones observed for black carbon (100–150 F gr^−1^) [48,49].

The association of the MMT with the low-temperature polypyrrole matrix with a slightly acidic medium improves the capacity and the electrochemical stability of the material. This is due to its good adsorption properties of charges on the MMT surface and their homogeneity, with a homogeneous distribution of the size of these pores, limiting the resistance effect (it is charged and discharged at a constant rate over the entire window of potential).

The cyclic voltammetric tests were performed with different scan rates on the NPC for the best ratio of MMT/PPy (Cl) (i.e., 0.4) (Figure 7). According to Zhang et al. [50], the low scanning rates allow diffusion of the electrolyte ions over the entire surface of the electrode, giving a rectangular shape to the voltammogram and a high specific capacitance. On the other hand, the increase in the scanning rate causes a deformation of the rectangular shape of the CV, therefore a decrease in the specific capacitance is observed. The same behavior was reported by Wang et al. in the case of the CCP MnO_2_/poly (aniline-CO-O-anisidine) [51,52].

**Figure 8:**
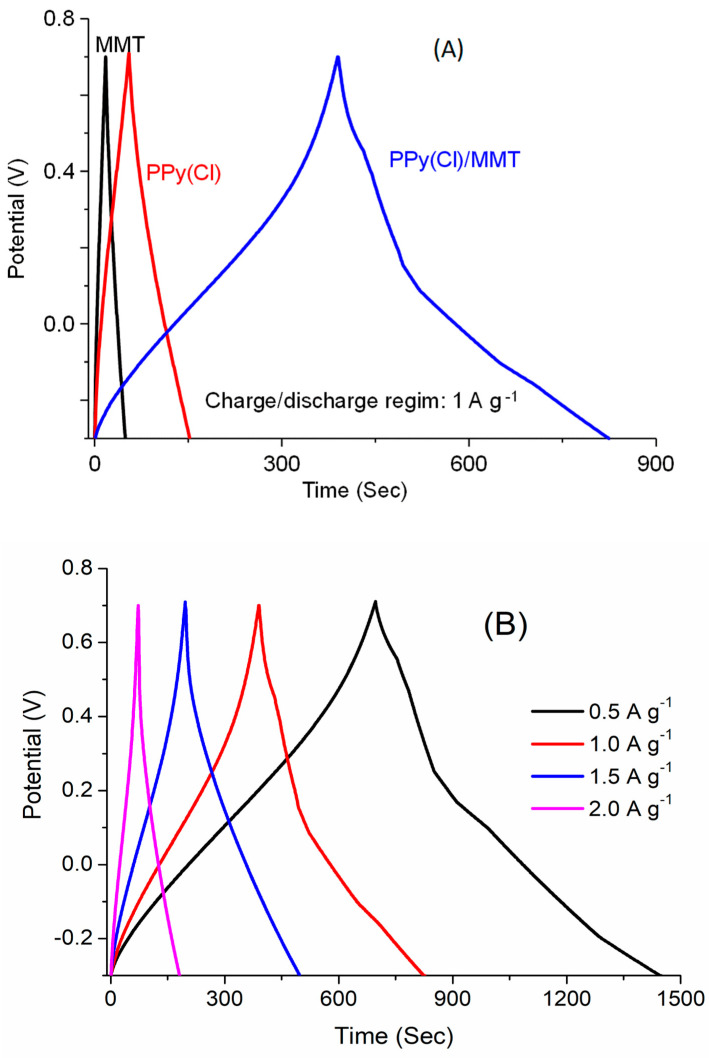
(**A**) Galvanostatic charge–discharge curves of MMT, PPy(Cl) and MMT/PPy(Cl) composite (mass ratio = 0.4) synthesized at 0 °C, (**B**) Effect of current density on the capacitive performance of MMT/PPy(Cl) composite.

The galvanostatic tests were carried out using a composite electrode based on 90% active material and 10% carbon black in an aqueous medium of H_2_SO_4_ 1 mol L^−1^. Figure 8A shows the profile of galvanostatic charge/discharge (GCD) processes for MMT, PPy and MMT/PPy(Cl) electrode materials at a current density of 1 A.g^−1^. The triangular shape of the PPy GCD curve suggests the presence of a faradaic pseudocapacitive behavior, while the slight deviation of the GCD curve of MMT/PPy(Cl) could be related to the existence of a faradaic redox reaction associated with a double layer capability. Similarly, the calculated specific capacitance of the MMT/PPy electrode composite (325 F g^−1^) is higher than that of PPy(Cl) (89 F g^−1^) when galvanostatically charged at 1 A g^−1^ [53,54]. Additionally, the GCD curves for the MMT/PPy(Cl) electrode composite at different current densities are shown in Figure 8B. It can be noted that the MMT/PPy(Cl) electrode composite exhibited 420, 270 and 180 F g^−1^ upon galvanostatic charging at 0.5–1.5 and 2 A g^−1^, respectively.

**Figure 9:**
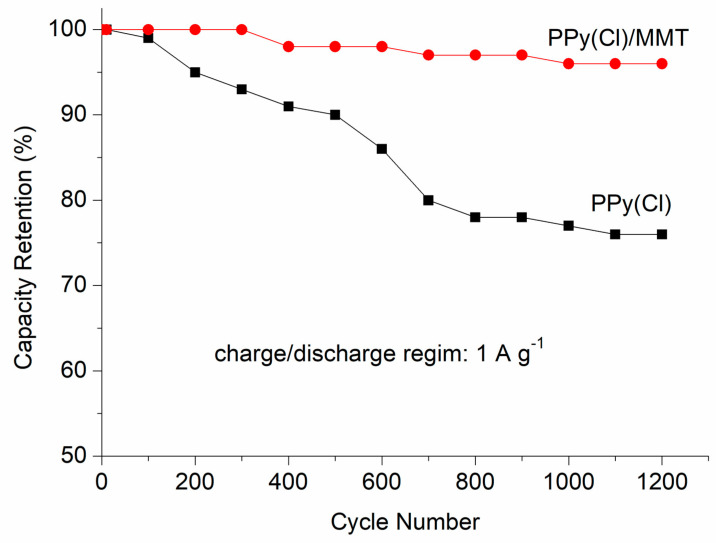
Cycle life performance of PPy(Cl) and MMT/PPy(Cl) composite (mass ratio = 0.4) composite.

Figure 9 shows a comparison of the cycling performance of PPy(Cl) with and without the addition of MMT. It was found that the capacity retention of PPy(Cl) quickly fades with increasing cycle count, reaching 75% at 1200 cycles. Conversely, the MMT/PPy(Cl) electrode composite showed 96% higher capacity retention after 1000 cycles, suggesting excellent cyclability.

**Figure 10:**
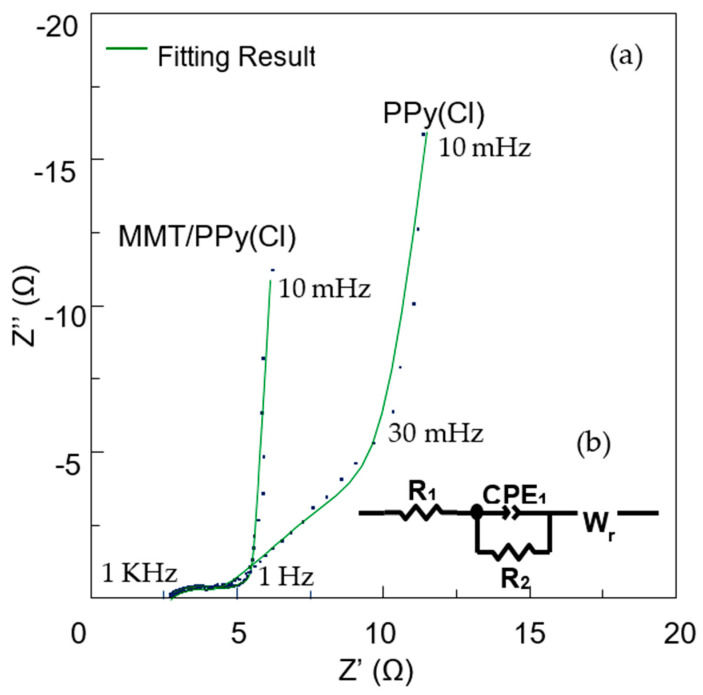
(**a**) Impedance spectra (Nyquist plot) between 1 kHz and 10 mHz of cells containing electrodes of PPy(Cl) and MMT/PPy(Cl) = 0.4. (**b**) equivalent circuit used for modelization.

Figure 10a displays impedance spectra of PPy(Cl) and MMT/PPy(Cl) (synthesized at 0 °C with 0.05 mol L^−1^ HCl), the measurements are carried out with cells with two symmetrical electrodes. The corner frequency, which is not very clear for MMT/PPy(Cl), appears at 30 mHz and separates two different phenomena in the cell: above this value, the frequency dependence of the real part of the impedance shows the penetration of the electrolyte into the porous structure of the electrode. Below this frequency, the impedance of the cell increases and tends to become purely capacitive. The frequency range below 30 mHz is related to electrolyte penetration into the porous structure [55,56]. At equal frequencies, the faster the ionic diffusion, the higher the slope (Ws (R) = 2.313 for MMT/PPy(Cl) compared to Ws (R) = 21.02 for PPy(Cl)). According to the impedance results obtained, it is also noticed that the diameter of the semicircle and the length of the line decrease by lowering the temperature of preparation of the material, which is probably due to the decrease in the transfer resistance charge and ion diffusion resistance [38,57]. Indeed, as we have seen previously, the presence of MMT gives a larger electro-active surface. It is also observed that the slope of the line at low frequencies increases for MMT/PPy(Cl), which shows that the synthesized composite is more capacitive than the polymer alone. However, we note that this line has a slope value less than 90°, which indicates the material does not exhibit an ideal behavior. This is presumably due to the heterogeneous distribution of pore sizes (CPE2 (α) = 0.455568 for MMT/PPy(Cl) compared to CPE2 (α) = 0.6004) for PPy(Cl)). These results are in good correlation with the results of cyclic voltammetry [8,58]. At very low frequencies, charged ions can propagate deep and even penetrate the scattering layer to produce a finite thickness Warburg (Wr) element. The experimental results of EIS are adjusted according to the appropriate equivalent circuit (Figure 10b) [59,60]. In the circuits, R_1_ is the so-called high-frequency electrolyte solution resistance [61]. The total impedance of the cell before cycling remains constant and, compared to that of the cell after cycling, the slope of the straight line in the low-frequency region does not show any change in the tilt angle, indicating excellent capacitive behavior and high cyclability of the composite electrode prepared at 0 °C in a medium containing 0.05 M mol.L^−1^ Cl.

## 4. Conclusions

This work investigated a low-cost natural clay with a layered structure and a large surface area used for electrochemical applications and energy storage. With the optimal temperature and the added HCl during the synthesis, a high capacity of 465 F g^−1^ can be achieved, competing with regular carbon-based composite materials. While lower temperatures seem to promote the adsorption of pyrrole onto the surface of montmorillonite, the impact of the added HCl can originate from the formation of side products that disrupt the conjugation within the polymer, reducing its conductivity. However, pyrrole adsorption can also be tuned by the ability of MMT to be protonated in an acid medium and lowering the monomer adsorption onto the clay surface before polymerization, respectively. By combining the optimized temperature and amount of HCl, the resulting composite showed a good stability while cycling. Although some loss of the capacity is observed (10–12%) after close to 100 cycles, the capacity remains constant for further cycles, showing that this kind of composite material can be involved in long-term electrochemical storage devices. Similarly, the calculated specific capacitance of the MMT/PPy electrode composite (325 F g^−1^) is higher than that of PPy(Cl) (89 F g^−1^) when galvanostatically charged at 1 A g^−1^.

## Data Availability

The data presented in this study are available on request from the corresponding author.

## Supplementary Materials

The following supporting information can be downloaded at: https://www.mdpi.com/article/10.3390/polym16070919/s1, Figure S1: SEM of (a) MMT and (b) MMT/PPy(Cl); Figure S2: Superposition of the XRD spectra of MMT and MMT/PPy(Cl); Figure S3: Electrical conductivity and specific capacity of MMT/PPy(Cl) synthesized at T = 0 °C with various concentrations of HCl used for the absorption of pyrrole on MMT; Figure S4: Direct optical transition of PPy(Cl), Figure S5: Direct optical transitions of MMT/PPy(Cl).

## References

[1] Zhang Y., Feng H., Wu X., Wang L., Zhang A., Xia T., Dong H., Li X., Zhang L. (2009). Progress of electrochemical capacitor electrode materials: A review. Int. J. Hydrogen Energy.

[2] Wang G., Zhang L., Zhang J. (2012). A review of electrode materials for electrochemical supercapacitors. Chem. Soc. Rev..

[3] Liu T., Finn L., Yu M., Wang H., Zhai T., Lu X., Tong Y., Li Y. (2014). Polyaniline and polypyrrole pseudocapacitor electrodes with excellent cycling stability. Nano Lett..

[4] Santino L.M., Lu Y., Acharya S., Bloom L., Cotton D., Wayne A., D’Arcy J.M. (2016). Enhancing Cycling Stability of Aqueous Polyaniline Electrochemical Capacitors. ACS Appl. Mater. Interfaces.

[5] Tian Y., Yang C., Song X., Liu J., Zhao L., Zhang P., Gao L. (2019). Engineering the volumetric effect of Polypyrrole for auto-deformable supercapacitor. Chem. Eng. J..

[6] Vishnuvardhan T.K., Basavaraja C., Raghavendra S.C. (2006). Synthesis, characterization and a.c. conductivity of polypyrrole/Y_2_O_3_ composites. Bull. Mater. Sci..

[7] Sivakkumar S.R., Ko J.M., Kim D.Y., Kim B.C., Wallace G.G. (2007). Performance evaluation of CNT/polypyrrole/MnO_2_ composite electrodes for electrochemical capacitors. Electrochim. Acta.

[8] Molahalli V., Bhat V.S., Shetty A., Hundekal D., Toghan A., Hegde G. (2023). ZnO doped SnO_2_ nano flower decorated on graphene oxide/polypyrrole nanotubes for symmetric supercapacitor applications. J. Energy Storage.

[9] Arvas M.B. (2023). Hydrothermal synthesis of polypyrrole/dye-functionalized carbon cloth electrode for wide potential window supercapacitor. Synth. Met..

[10] Ramasamy V., Sathishpriya T., Thenpandiyan E., Suresh G., Sagadevan S. (2023). Short communication A facile and eco-friendly synthesis of Mn-doped CaCO_3_/PMMA nanocomposite for highly efficient supercapacitor in energy storage applications. Inorg. Chem. Commun..

[11] Xiao S., Bi F., Zhao L., Wang L., Gai G. (2017). Design and synthesis of H-TiO_2_/MnO_2_ core–shell nanotube arrays with high capacitance and cycling stability for supercapacitors. J. Mater. Sci..

[12] Al-hamyd M.A., Al-asadi A.S., Al-mudhaffer M.F. (2022). Physica E: Low-dimensional Systems and Nanostructures Preparation and characterization of zinc–aluminum layered doubled hydroxide/graphene nanosheets composite for supercapacitor electrode. Phys. E Low-Dimens. Syst. Nanostructures.

[13] Mazeikiene R., Malinauskas A. (2002). Kinetics of the electrochemical degradation of polypyrrole. Polym. Degrad. Stab..

[14] Zhang W., Ren Z., Ying Z., Liu X., Wan H. (2018). Activated nitrogen-doped porous carbon ensemble on montmorillonite for high-performance supercapacitors. J. Alloys Compd..

[15] Jlassi K., Singh A., Aswal D.K., Losno R., Benna-Zayani M., Chehimi M.M. (2013). Novel, ternary clay/polypyrrole/silver hybrid materials through in situ photopolymerization. Colloids Surf. A Physicochem. Eng. Asp..

[16] Maiti S., Pramanik A., Chattopadhyay S., De G., Mahanty S. (2016). Electrochemical energy storage in montmorillonite K10 clay based composite as supercapacitor using ionic liquid electrolyte. J. Colloid Interface Sci..

[17] Gierszewska M., Jakubowska E., Olewnik-Kruszkowska E. (2019). Effect of chemical crosslinking on properties of chitosan-montmorillonite composites. Polym. Test..

[18] Ge W., Ma Q., Ai Z., Wang W., Jia F., Song S. (2021). Applied Clay Science Three-dimensional reduced graphene oxide/montmorillonite nanosheet aerogels as electrode material for supercapacitor application. Appl. Clay Sci..

[19] Luo X., Hsu F., Gan Y., Pao C., Lee M., Wang C., Lin J., Chen C., Wu K., Chuang W. (2023). Intercalation of Fe-montmorillonite for developing nacre-inspired flexible all-solid-state supercapacitor with circular economy approach. Chin. J. Phys..

[20] Tian W., Mao X., Brown P., Rutledge G.C., Hatton T.A. (2015). Electrochemically Nanostructured Polyvinylferrocene/Polypyrrole Hybrids with Synergy for Energy Storage. Adv. Funct. Mater..

[21] Kulandaivalu S., Mohd Azahari M.N., Azman N.H.N., Sulaiman Y. (2020). Ultrahigh specific energy of layer by layer polypyrrole/graphene oxide/multi-walled carbon nanotube| polypyrrole/manganese oxide composite for supercapacitor. J. Energy Storage.

[22] Vijeth H., Ashokkumar S.P., Yesappa L., Vandana M., Devendrappa H. (2020). Nickel oxide nanoparticle incorporated polypyrrole nanocomposite for supercapacitor application. AIP Conf. Proc..

[23] Kim S.H., Kim Y.I., Park J.H., Ko J.M. (2009). Cobalt-manganese oxide/carbon-nanofiber composite electrodes for supercapacitors. Int. J. Electrochem. Sci..

[24] Zhu J., Feng T., Du X., Wang J., Hu J., Wei L.P. (2017). High performance asymmetric supercapacitor based on polypyrrole/graphene composite and its derived nitrogen-doped carbon nano-sheets. J. Power Sources.

[25] Botana A., Mollo M., Eisenberg P., Sanchez R.M.T. (2010). Applied Clay Science Effect of modi fi ed montmorillonite on biodegradable PHB nanocomposites. Appl. Clay Sci..

[26] Mejri C., Oueslati W., Ben A., Amara H. (2023). Applied Surface Science Advances Structure and reactivity assessment of dioctahedral montmorillonite during provoked variable sequential cation exchange process via XRD modelling approach. Appl. Surf. Sci. Adv..

[27] Boudjema J.M.F.S., Vispe E., Choukchou-Braham A., Mayoral J.A., Bachir R. (2015). Preparation and characterization of activated montmorillonite clay supported 11-molybdo vanadophosphoric acid for cyclohexene oxidation. RSC Adv..

[28] Khatti T., Naderi-manesh H., Mehdi S. (2019). Materials Science & Engineering B Polypyrrole-Coated Polycaprolactone-Gelatin Conductive Nano fi bers: Fabrication and Characterization. Mater. Sci. Eng. B.

[29] Varesano A., Dall’Acqua L., Tonin C. (2005). A study on the electrical conductivity decay of polypyrrole coated wool textiles. Polym. Degrad. Stab..

[30] Farbod M., Mobini N. (2014). Physical properties, thermal stability, and glass transition temperature of multi-walled carbon nanotube/polypyrrole nanocomposites. Compos. Interfaces.

[31] Belabed C., Haine N., Benabdelghani Z., Bellal B., Trari M. (2014). Photocatalytic hydrogen evolution on the hetero-system polypyrrol/TiO_2_ under visible light. Int. J. Hydrogen Energy.

[32] Wang S., Zhou Y., Liu Y., Wang L., Gao C. (2020). Enhanced thermoelectric properties of polyaniline/polypyrrole/carbon nanotube ternary composites by treatment with a secondary dopant using ferric chloride. J. Mater. Chem. C.

[33] Borralleras P., Segura I., Aranda M.A.G., Aguado A. (2019). Influence of the polymer structure of polycarboxylate-based superplasticizers on the intercalation behaviour in montmorillonite clays. Constr. Build. Mater..

[34] Yeh J.M., Chin C.P., Chang S. (2003). Enhanced corrosion protection coatings prepared from soluble electronically conductive polypyrrole-clay nanocomposite materials. J. Appl. Polym. Sci..

[35] Kuila B., Nandi A. (2006). Structural hierarchy in melt-processed poly (3-hexyl thiophene)-montmorillonite clay nanocomposites: Novel physical, mechanical, optical, and conductivity properties. J. Phys. Chem. B.

[36] Li N., Yang B., Xu L., Xu G., Sun W., Yu S. (2016). Simple synthesis of Cu_2_O/Na-bentonite composites and their excellent photocatalytic properties in treating methyl orange solution. Ceram. Int..

[37] Olad A., Rashidzadeh A., Amini M. (2013). Preparation of Polypyrrole Nanocomposites with Organophilic and Hydrophilic Montmorillonite and Investigation of Their Corrosion Protection on Iron. Adv. Polym. Technol..

[38] Guo C., Tian S., Chen B., Liu H., Li J. (2020). Constructing α-MnO_2_@PPy core-shell nanorods towards enhancing electrochemical behaviors in aqueous zinc ion battery. Mater. Lett..

[39] Dzulkurnain N.A., Mokhtar M., Rashid J.I.A., Knight V.F., Wan Yunus W.M.Z., Ong K.K., Mohd Kasim N.A., Mohd Noor S.A. (2021). A review on impedimetric and voltammetric analysis based on polypyrrole conducting polymers for electrochemical sensing applications. Polymers.

[40] Vilímová P., Kulhánková L., Peikertová P., Mamulová Kutláková K., Vallová S., Koníčková H., Plaček T., Tokarský J. (2019). Effect of montmorillonite/polypyrrole ratio and oxidizing agent on structure and electrical conductivity of intercalated nanocomposites. Appl. Clay Sci..

[41] Gao J.W., Li G., Yao Y.F., Jiang J.M. (2011). Preparation and characterization of montmorillonite/polypyrrole nanocomposites by in-situ chemical polymerizatio. J. Macromol. Sci. Part B Phys..

[42] Churchman G.J., Jackson M.L. (1976). Reaction of montmorillonite with acid aqueous solutions: Solute activity control by a secondary phase. Geochim. Cosmochim. Acta.

[43] Liu X., Lu X., Sprik M., Cheng J., Meijer E.J., Wang R. (2013). Acidity of edge surface sites of montmorillonite and kaolinite. Geochim. Cosmochim. Acta.

[44] Brady P.V., Cygan R.T., Nagy K.L. (1996). Molecular controls on kaolinite surface charge. J. Colloid Interface Sci..

[45] Ramoa S.D.A., Barra G.M.O., Merlini C., Livi S., Soares B.G., Pegoretti A. (2015). Novel electrically conductive polyurethane/montmorillonite-polypyrrole nanocomposites. Express Polym. Lett..

[46] Rapi S., Bocchi V., Gardini G.P. (1988). Conducting polypyrrole by chemical synthesis in water. Synth. Met..

[47] Garsuch A., Sattler R.R., Pickup P.G. (2004). Formation of polypyrrole from 2,5-bis(2-pyrrolyl)pyrrolidine. Chem. Commun..

[48] Sun Z., Thielemans W. (2023). Interconnected and high cycling stability polypyrrole supercapacitors using cellulose nanocrystals and commonly used inorganic salts as dopants. J. Energy Chem..

[49] Wang Y., Du Pasquier A., Li D., Atanassova P., Sawrey S., Oljaca M. (2018). Electrochemical double layer capacitors containing carbon black additives for improved capacitance and cycle life. Carbon.

[50] Zhang J., Kong L.-B., Cai J.-J., Luo Y.-C., Kang L. (2010). Nano-composite of polypyrrole/modified mesoporous carbon for electrochemical capacitor application. Electrochim. Acta.

[51] Yang X.-F., Wang G.-C., Wang R.-Y., Li X.-W. (2010). Key A novel layered manganese oxide/poly(aniline-co-o-anisidine) nanocomposite and its application for electrochemical supercapacitor. Electrochim. Acta.

[52] Abdillah O.B., Rus Y.B., Ulfa M., Dedi, Iskandar F. (2023). Recent progress on reduced graphene oxide and polypyrrole composites for high performance supercapacitors: A review. J. Energy Storage.

[53] Yang K., Cho K., Kim S. (2018). Effect of carbon black addition on thermal stability and capacitive performances of supercapacitors. Sci. Rep..

[54] Fahim H., Sanad M.M.S., Ghebache Z., Boudieb N. (2022). Effect of polymerization conditions on the physicochemical and supercapacitor applications electrochemical properties of SnO_2_/polypyrrole composites for supercapacitor applications. J. Mol. Struct..

[55] Pandolfo A.G., Hollenkamp A.F. (2006). Carbon properties and their role in supercapacitors. J. Power Sources.

[56] Yang Y., Xiao R., Sun X., Lu L., Chen Y. (2022). Constructing two dimensional composite nanosheets with montmorillonite and graphene-like carbon: Towards high-rate-performance PVA based gel polymer electrolytes for quasi-solid-state supercapacitors. Mater. Chem. Phys..

[57] Wang J.G., Yang Y., Huang Z.H., Kang F. (2012). Interfacial synthesis of mesoporous MnO_2_/polyaniline hollow spheres and their application in electrochemical capacitors. J. Power Sources.

[58] Torabi M., Soltani M., Sadrnezhaad S.K. (2014). Impedance analysis of growth and morphology of electropolymerized polypyrrole nanocomposites. J. New Mater. Electrochem. Syst..

[59] Zhu S., Sun X., Gao X., Wang J., Zhao N., Sha J. (2019). Equivalent circuit model recognition of electrochemical impedance spectroscopy via machine learning. J. Electroanal. Chem..

[60] Sanqing H., Dejin B., Yanfei X., Huijuan L. (2023). Facile Construction of Three-Dimensional Architectures of a Nanostructured Polypyrrole on Carbon Nanotube Fibers and Their Effect on Supercapacitor Performance. ACS Appl. Energy Mater..

[61] Hallik A., Alumaa A., Tamm J., Sammelselg V., Väärtnõu M., Jänes A., Lust E. (2006). Analysis of electrochemical impedance of polypyrrole|sulfate and polypyrrole|perchlorate films. Synth. Met..

