# Supplementary material for: Montmorillonite/Poly(Pyrrole) for Low-Cost Supercapacitor Electrode Hybrid Materials
Source: Polymers (Basel). 2024 Mar 27;16(7):919. doi: 10.3390/polym16070919 (PMC11013034; doi:10.3390/polym16070919)
Supplement: Supplementary file 1 [file polymers-16-00919-s001.zip › polymers-2805748-supplementary.pdf]

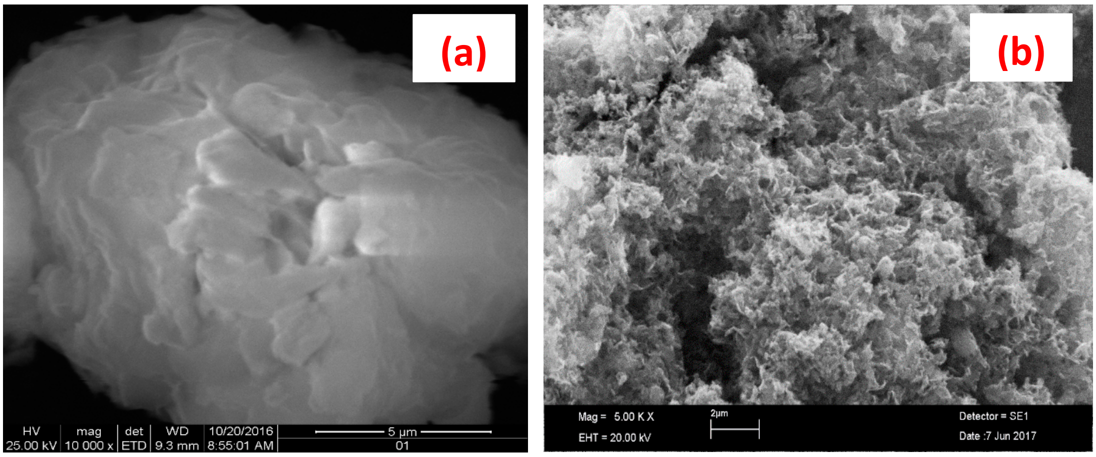

Figure S1: SEM of (a) MMT and (b) MMT/PPy(Cl)

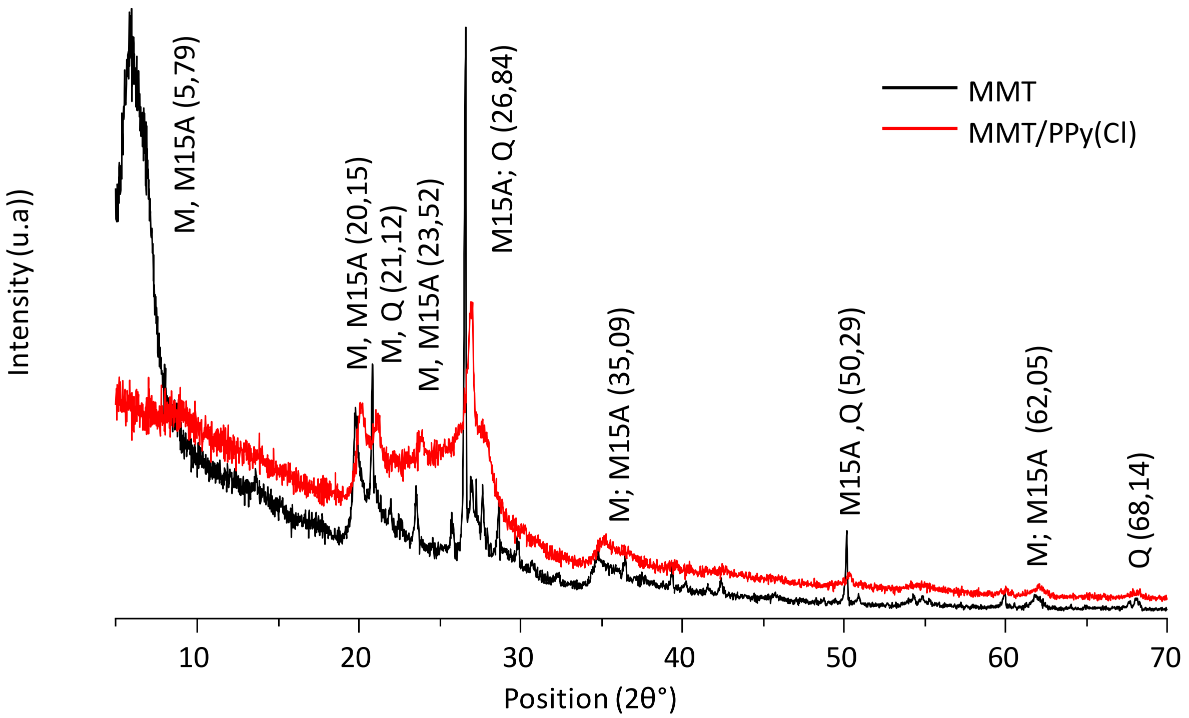

Figure S2: Superposition of the XRD spectra of MMT and MMT/PPy(Cl)

M: Montmorillonite ($Si_{3.74}Al_{2.03}Fe_{0.03}Mg_{0.02}O_{11}$)

M15A: Montmorillonite 15A ($Ca_{0.2}$ (Al, Mg) $2Si_4O_{10}$ $(OH)_2 \cdot 4H_2O$)

Q: Quartz (SiO_2)

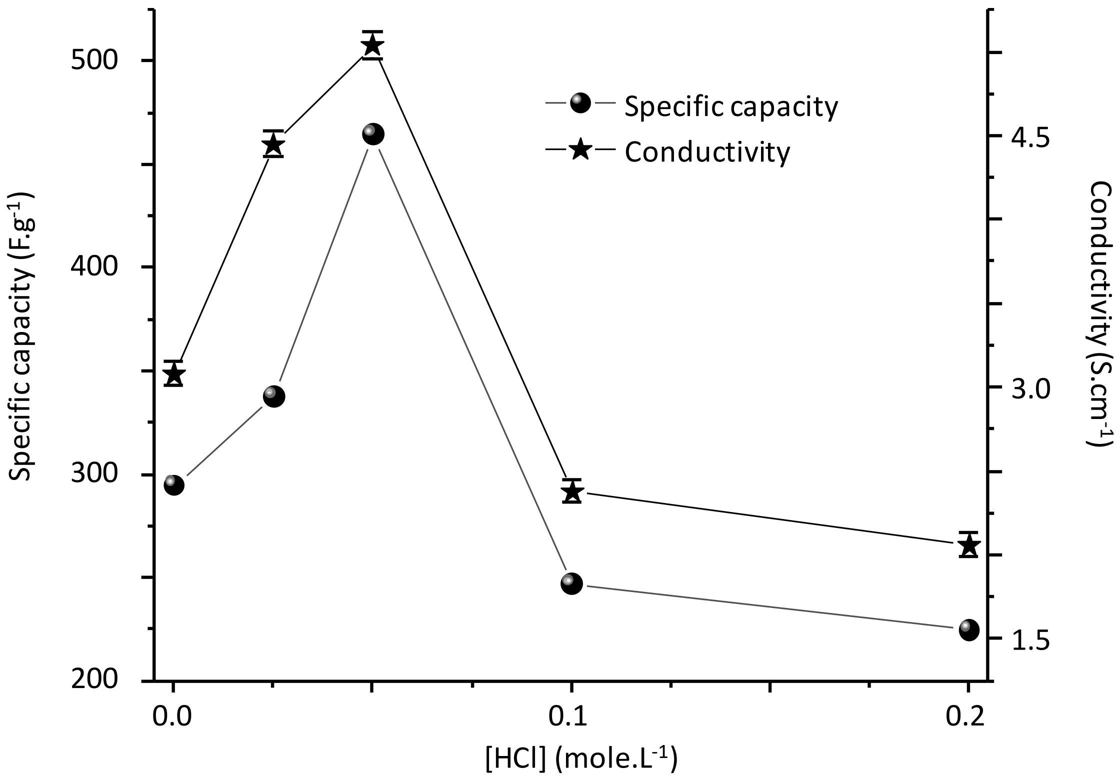

Figure S3: Electrical conductivity and specific capacity of MMT/PPy(Cl) synthesized at T=0°C with various concentrations of HCl used for the absorption of pyrrole on MMT

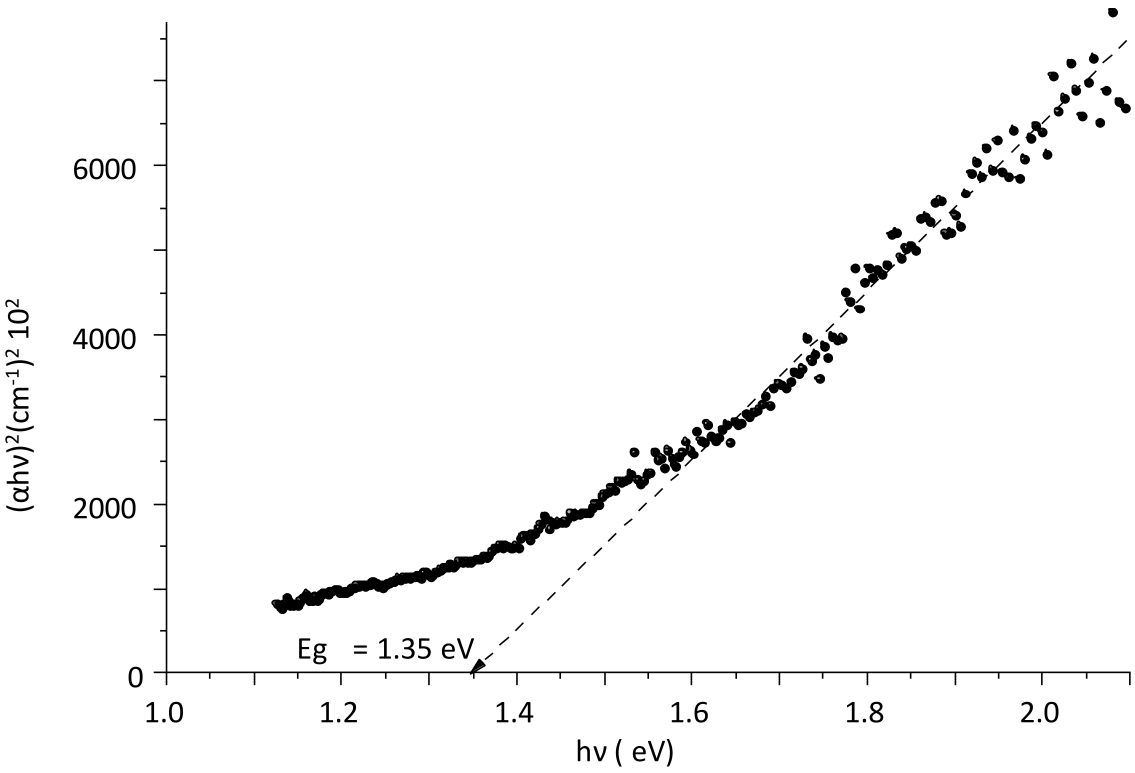

Figure S4: Direct optical transition of PPy(Cl)

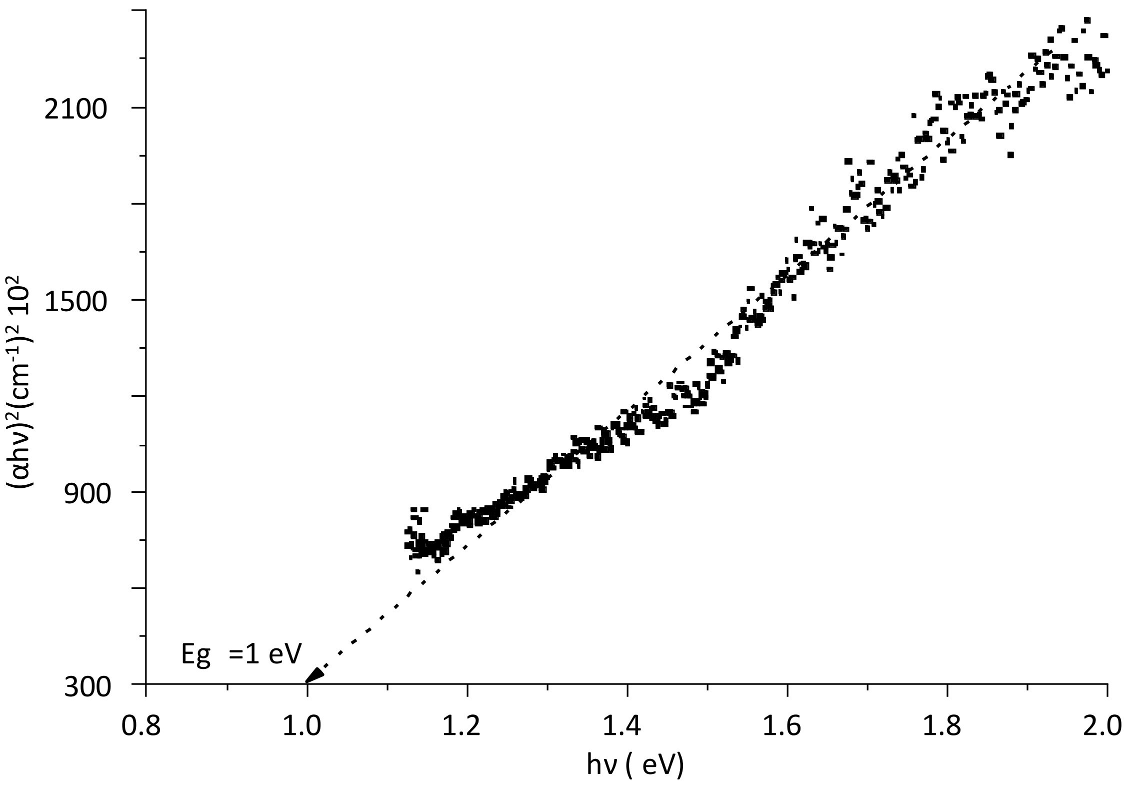

Figure S5: Direct optical transitions of MMT/PPy(Cl)
